# Special Hospitals and Their Advertisements

**Published:** 1912-06-15

**Authors:** 


*To the Editor of* The Hospital.

### Special Hospitals and their Advertisements.

*To the Editor of* The Hospital.

Sir,—From experience a little similar to Mr. Forrest's as described in your last issue, I can endorse some of his statements, but not others.

In the first place, I should judge that, speaking generally, Metropolitan throat and ear hospitals attract a better-off class of patients than do general ones. An out-patient sister (no bad judge of the situation) at one of them said once to me, "Why, some of our patients pay as much as five shillings a visit! If they come in and occupy a bed they always pay something—in fact, we are never in debt." This has doubtless something to do with the fact that, year after year, the Royal Fund makes a grant to several special hospitals of this kind, conditionally upon their amalgamating; and the said special hospitals in effect snap their fingers in reply, and go on as they have been doing. But I should doubt much Mr. Forrest's assertion that the motive is to keep oto-rhino-laryngology from the general practitioner. If it is, why do all of them, and especially the particular one he names, make such a feature of post-graduate instruction? I never saw an instance of a hospital surgeon trying to get a private patient from his out-patient clinic except once, and in that case he offered him the choice of the whole staff. The man was well able to pay—in fact, it turned out he owned several public-houses. But he refused the operation, so that was the end of it. I doubt, too, whether many general practitioners are competent to keep abreast of modern laryngology. On the one hand, the outlets for their skill and energy are so numerous : nowadays general practice demands far too much of a man. On the other, their cases would be so few that it would not pay them to devote the time to acquiring the necessary knowledge or the money for buying expensive special instruments. One must be in touch with some sort of special clinic, say, at a smaller provincial infirmary, to get enough clinical material to make the enterprise worth while.

If it be true that special hospitals advertise in lay newspapers, this certainly should be stopped at once.—Yours faithfully, Epiglottis.

